# The Association between Habitual Sleep Duration and Sleep Quality with Glycemic Traits: Assessment by Cross-Sectional and Mendelian Randomization Analyses

**DOI:** 10.3390/jcm8050682

**Published:** 2019-05-15

**Authors:** Maxime M. Bos, Diana van Heemst, Esther Donga, Renée de Mutsert, Frits R. Rosendaal, Gerard Jan Blauw, Patrick C. N. Rensen, Nienke R. Biermasz, Raymond Noordam

**Affiliations:** 1 Department of Internal Medicine, section of Gerontology and Geriatrics, Leiden University Medical Center, P.O. Box 9600, 2300 RC Leiden, The Netherlands; d.van_heemst@lumc.nl (D.v.H.); g.j.blauw@lumc.nl (G.J.B.); 2 Departments of Radiology and Internal Medicine, Elisabeth TweeSteden Hospital, Hilvarenbeekseweg 60, 5022 GC Tilburg, The Netherlands; e.donga@etz.nl; 3 Department of Clinical Epidemiology, Leiden University Medical Center, P.O. Box 9600, 2300 RC Leiden, The Netherlands; r.de_mutsert@lumc.nl (R.d.M.); f.r.rosendaal@lumc.nl (F.R.R.); 4 Einthoven Laboratory for Experimental Vascular Medicine, Leiden University Medical Center, P.O. Box 9600, 2300 RC Leiden, The Netherlands; p.c.n.rensen@lumc.nl; 5 Department of Medicine, Division of Endocrinology, Leiden University Medical Center, P.O. Box 9600, 2300 RC Leiden, The Netherlands; n.r.biermasz@lumc.nl

**Keywords:** sleep, diabetes, epidemiology, insulin resistance, mendelian randomization

## Abstract

Evidence on whether habitual sleep duration and sleep quality are associated with increased insulin resistance is inconsistent. Here, we investigated the associations between different measures of habitual sleep with glycemic traits through cross-sectional and Mendelian randomization (MR) analyses. We assessed the associations of sleep duration and sleep quality with glycemic traits using multivariable linear regression models adjusted for potential confounders in 4672 middle-aged (45–65 years; 48% men) nondiabetic participants of the Netherlands Epidemiology of Obesity (NEO) study. Genetic variants for total, short, and long sleep duration were used as instrumental variables in MR analyses using summary-level data of glycemic traits in nondiabetic individuals (MAGIC; *n* = 58,074). In cross-sectional analyses, shortest sleepers (median 5.0 h of sleep per night) had 14.5% (95% confidence interval (CI): 2.0; 28.6%) higher fasting insulin level and 16.3% (95% CI: 2.7; 31.7%) higher HOMA-β. Bad sleep quality was associated with higher insulin resistance (e.g., 14.3% (95% CI: 4.7; 24.9%) higher HOMA-IR). All these associations disappeared after adjustment for BMI and the risk of sleep apnea. MR analyses did not indicate a causal association between total, short or long sleep duration and glycemic traits. Therefore, our used measures of habitual sleep duration and sleep quality are unlikely to directly associate with insulin resistance.

## 1. Introduction

During the past few decades, obesity and conditions that reflect disturbances in metabolism have increased [1]. Multiple studies have assessed the association between measures of habitual sleep and metabolic diseases. In several studies, it has been observed that both short and long total sleep duration were associated with a higher risk of obesity, insulin resistance, diabetes mellitus, and a higher body weight [2,3,4,5,6,7,8,9]. However, other studies reported only short sleep duration and not long sleep duration to be associated with a higher risk of obesity and metabolic syndrome [6,10,11]. As total sleep duration has decreased over the past decades [12], studies in this area are of increasing importance. 

Several factors could contribute to the discrepancy in the observed associations between sleep duration and insulin resistance. For example, most studies investigated only short sleep duration [2], or short sleep duration under artificial circumstances [2,13], which might not resemble habitual short sleep duration. Moreover, cut-offs to define either short or long sleep duration are heterogeneous [14]. Another explanation might be adjustments for confounding factors. Body mass index (BMI) is known on one hand to be one of the largest risk factors for insulin resistance and diabetes, while on the other hand, it is associated with shorter sleep duration [15,16,17]. In a study in adolescents, an association between shorter sleep duration and insulin resistance was observed; however, this association disappeared after adjustment for BMI [18]. Although related to BMI, another potential confounding factor is the presence of obstructive sleep apnea (OSA). The presence of OSA was demonstrated to be associated with a higher risk of insulin resistance and diabetes [19]. However, most of the previous performed studies did not adjust for OSA [4,10,11,20,21,22].

The question remains as to what extent the previously described associations between sleep duration with insulin resistance were confounded by BMI and OSA. In addition to sleep duration, poor sleep quality is associated with a higher presence of obesity, metabolic syndrome, and diabetes [23,24]. However, studies examining poor sleep quality have mainly been performed in patients with diabetes [5,24,25]. If assessed in nondiabetic individuals, artificially altered sleep quality was studied by, e.g., suppressing slow-wave sleep [26], which does not resemble habitual poor sleep quality. Moreover, methods to assess sleep quality differed between studies. For example, some studies used only a single question about difficulty of initiating or maintaining sleep [5], while others used the Pittsburgh sleep quality index (PSQI) questionnaire [25] or other questionnaires [27,28]. These different methods to assess sleep quality complicate comparability among these studies. 

Based on earlier studies, we hypothesized that both short sleep duration and bad sleep quality are associated with higher insulin resistance, but this association is likely dependent on anthropometric traits like BMI and the risk of sleep apnea. We aimed to examine this hypothesis in a middle-aged nondiabetic population embedded in the Netherlands Epidemiology of Obesity (NEO) study [29]. Furthermore, we extended this study with a two-sample Mendelian randomization (MR) analysis to provide evidence as to whether the association between sleep duration and insulin resistance is causal using data of the Meta-Analyses of Glucose and Insulin-Related Traits Consortium (MAGIC). 

## 2. Experimental Section

### 2.1. Study Design and Study Population

The Netherlands Epidemiology of Obesity (NEO) study is a population-based cohort study. Participants were recruited from September 2008 until September 2012, resulting in a cohort of 6671 individuals, with an oversampling of individuals with overweight or obesity. Men and women aged between 45 and 65 years with a self-reported body mass index (BMI) of 27 kg/m^2^ or higher living in the greater area of Leiden were eligible to participate in the NEO study. In addition, all inhabitants aged between 45 and 65 years from one municipality (Leiderdorp) were invited irrespective of their BMI, allowing for a reference distribution of BMI. Baseline data were collected at the NEO study center of the Leiden University Medical Center (LUMC). Prior to the NEO study visit, participants completed a questionnaire about demographic and clinical information and fasted for at least 10 h. Participants came to the research site in the morning to undergo several baseline measurements, including anthropometric measurements and blood sampling. The participants drank a liquid mixed meal, after which postprandial blood sampling was performed. All medication used in the month preceding the visit to the study center was recorded by research nurses. More detailed information on the study design and data collection has been described elsewhere [29]. The NEO study was approved by the medical ethics committee of the LUMC (P08.109), and has been registered under NCT03410316 (https://clinicaltrials.gov). All participants provided written informed consent. 

In the present study, we excluded participants with missing data on the PSQI questionnaire (*n* = 1402), which were collected in participants enrolled in NEO after July 2009. Moreover, we excluded participants that used glucose lowering medication (*n* = 263), had a medical history of diabetes mellitus (*n* = 77), had missing baseline characteristics (*n* = 132), had missing glucose or insulin concentrations (*n* = 48), had missing data on the Berlin questionnaire (*n* = 57) or did not fast (*n* = 20). In the analyses with the postprandial measures, we additionally excluded participants with incomplete or no liquid meal intake (*n* = 8) and participants with either missing postprandial glucose (*n* = 153) or postprandial insulin concentrations (*n* = 78). 

### 2.2. Sleep Characteristics

To assess habitual sleep duration and sleep quality, we used data collected with the PSQI [30], which is a self-rated questionnaire, to retrospectively measure sleep parameters over a one-month time period. Total sleep duration was derived from the question: “On an average night, how much sleep do you get?” To obtain a classification of (extreme) short and long total sleep duration independent of age and sex, we calculated the age- and sex-adjusted residuals with linear regression analysis for total sleep duration and determined subgroups on the basis of these residuals, as previously described [17,31]. We used the 5th lowest percentile to define shortest sleep, the 5th to the 20th percentile to define short sleep, the 20th to the 80th to define medium sleep, the 80th to the 95th to define long sleep, and the 95th to the 100th percentile to define longest sleep of the adjusted residuals. Sleep quality was assessed using the total score of the PSQI questionnaire. The questionnaire consists of seven components, based on which an overall score can be calculated ranging from 0 to 21, in which a higher score indicates poorer sleep quality [30]. In the sleep quality analyses, the good sleep quality group (PSQI total score ≤5) was used as a reference group in linear regression analyses with two groups with either a PSQI total score of >5 or ≥10, the latter in order to investigate a more extreme disturbed habitual sleep. 

### 2.3. Glycemic Traits

After an overnight fast of at least 10 h, fasting blood samples were taken at the study center. Within 5 m after the first blood sample was taken, participants drank a liquid mixed meal. This meal (400 mL) contained 600 kcal, with 16% of energy derived (En%) from protein, 50% En% from carbohydrates, and 34 En% from fat. Two postprandial blood samples were taken at 30 and 150 m after ingestion of the mixed meal. Serum and plasma were collected during each of the three blood draws, and concentrations of glucose and insulin were determined. Fasting plasma glucose concentrations were determined by enzymatic and colorimetric methods (Roche Modular Analytics P800, Roche Diagnostics, Mannheim, Germany; CV < 5%), and serum insulin concentrations were determined by an immunometric method (Siemens Immulite 2500, Siemens Healthcare Diagnostics, Breda, The Netherlands; CV < 5%). All analyses were performed in the central clinical chemistry laboratory of the Leiden University Medical Center. Fasting glucose and insulin levels were used to calculate the homeostatic model assessment of insulin resistance (HOMA-IR) index as a marker for hepatic insulin resistance. The HOMA-IR was calculated using (fasting insulin × fasting glucose)/22.5. HOMA of β-cell function (HOMA-β) was used as a marker that indicates basal insulin release and calculated as 20 × (fasting insulin/fasting glucose) − 3.5 [32,33]. The area under the curve (AUC) for postprandial overall glucose and insulin levels was calculated using the trapezoid rule as (15 × fasting concentration + 75 × concentration_30min_ + 60 × concentration_150min_)/150 [34]. 

### 2.4. Covariates

Level of education was reported in 10 categories according to the Dutch education system and grouped into high versus low education [29]. A semiquantitative food frequency questionnaire (FFQ) [35] questionnaire was used to assess daily total energy intake. Energy intake was estimated from the FFQ with the 2011 version of the Dutch food composition table (NEVO-2011). Participants reported the frequency and duration of their physical activity in leisure time using the short questionnaire to assess health-enhancing physical activity (SQUASH) [36], which was expressed in hours per week of metabolic equivalents (MET-h/week). Body weight was measured without shoes, and one kilogram (kg) was subtracted to correct for the weight of clothing. BMI was calculated by dividing the weight in kilograms by the height in meters squared. The Berlin questionnaire was used to assess the risk for the presence of obstructive sleep apnea syndrome [37]. This questionnaire consists of 10 questions that form three categories (snoring (category 1), daytime somnolence (category 2), and hypertension and BMI (category 3)) related to the likelihood of the presence of sleep apnea. Individuals can be classified as either having a high (2 or more categories with a positive score) or low likelihood of the presence of sleep apnea (only 1 or no categories with a positive score). 

### 2.5. Statistical Analysis

Because individuals with a BMI of 27 kg/m^2^ or higher were oversampled in the NEO study, adjustments were made to correctly represent associations in the general population [38]. This was done by weighting individuals towards the BMI distribution of participants from the Leiderdorp municipality, whose BMI distribution was similar to the BMI distribution of the general Dutch population. All presented results are based on weighted analyses. Consequently, the results apply to a population-based study without oversampling of participants with a BMI of 27 kg/m^2^ or higher. We performed all statistical analyses of the NEO cohort using Stata version 12.1 (Stata, College Station, TX, USA).

Baseline characteristics were expressed as means (with standard deviation, SD) for normally distributed measures, median with interquartile ranges for non-normally distributed measures, and proportions for categorical variables. 

Not normally distributed outcomes were log-transformed to approximate a normal distribution (notably fasting insulin, HOMA-IR, HOMA-β, AUC insulin). In order to present the results with a similar interpretation, normally distributed outcomes (notably fasting glucose and AUC glucose) were log-transformed as well. All outcome variables were approximately normally distributed after log-transformation, including those that were already normally distributed prior to the transformation. Linear regression analyses using the medium sleep category (characterized by the 20th to the 80th percentile of sleep duration residuals) as a reference group were performed. The subsequent beta regression coefficients are expressed as percentages with accompanying 95% confidence interval (95% CI), which can be interpreted as the percentage change in outcome with respect to the reference group. The initial model in linear regression analyses was adjusted for age and sex (Model 1). In addition to age and sex, we adjusted Model 2 for ethnicity (white/other), education level (high/other), smoking (never/former/current), alcohol consumption (g/day), energy intake (kJ/day), physical activity (MET/h/week), and sleep medication (yes/no). In Model 3, we additionally adjusted for sleep apnea (high risk/low risk) and BMI (kg/m^2^). All selected covariables were only low to moderately correlated with each other (Supplementary Figure S1). In the analyses for sleep quality, we did not adjust for sleep medication in Models 2 and 3, as this is a component of the PSQI total score.

### 2.6. Mendelian Randomization Analysis

For the MR analysis, we selected 78 single nucleotide polymorphisms (SNPs) that have been shown to associate with self-reported total sleep duration (*p*-value < 5e^−8^) as genetic instruments from the largest genome-wide association study (GWAS; source: UK Biobank, 446,118 unrelated European-ancestry individuals) [39] (Supplementary Table S4). Moreover, we selected 27 SNPs associated with short sleep duration (<7 h; *n* = 106,192 cases) and 8 SNPs associated with long sleep duration (≥9 h; *n* = 34,184 cases) relative to 7–8 h of sleep duration (*n* = 305,742) (Supplementary Tables S5 and S6). 

Summary statistics data of GWAS on glycemic traits were used as outcomes in a two-sample MR approach. We used data from MAGIC, which comprised a meta-analysis of European ancestry studies that investigated genetic variants associated with glycemic traits. For fasting glucose and fasting insulin, we used the GWAS of Manning et al. (2012) [40], which included a total of 58,074 and 51,570 individuals without diabetes mellitus, respectively. In addition, we used HOMA-IR and HOMA-B as measures of insulin resistance (*n* = 46,186) [41]. 

For the two-sample MR analyses, we used similar methodology as described previously [42]. In short, we combined effects of the individual genetic instruments using the inverse variance weighted (IVW) approach as our main analysis method with MRCIEU/TwoSampleMR package in R [43]. The resulting estimate (presented with accompanying 95% CI) can be interpreted as a weighted mean effect of a genetically determined increase in total sleep duration (per hour) and a higher risk for short or long sleep duration on our study outcomes. In order to formally test for potential pleiotropic effects of the genetic variants, in which the genetic variants have pleiotropic effects that influence glycemic traits via alternative pathways, we conducted MR–Egger regression as sensitivity analysis [44]. We furthermore conducted weighted median estimator (WME) analyses; similarity between the IVW and WME effect estimates is indicative of robustness of the results [45]. 

## 3. Results

### 3.1. Baseline Characteristics

In the present study, we included a total of 4672 participants with a mean age of 56 (SD 6.0) years, of whom 48% were men. In Table 1, the population characteristics are presented for the study population, stratified by the sleep duration groups. As compared to the medium sleep group (sleep duration = 7 h/day), participants in the shortest sleep group (sleep duration = 5 h/day) were more of nonwhite ancestry (96% vs. 90%), had a lower education (50% high vs. 39% high), higher BMI (26 vs. 27 kg/m^2^), smoked more (16% vs. 18%), were less physically active (31 vs. 25 MET/h/week), used more sleep medication (4% vs. 14%), had a higher PSQI total score (4 vs. 11), and had a higher risk of having sleep apnea (17% vs. 35%). In the shortest sleep group, fasting insulin, HOMA-β, and AUC of insulin were higher than in the medium sleep group. As compared to the medium group, the longest sleep duration group consisted of more men (42% vs. 53%), more current smokers (16% vs. 20%), fewer high-educated participants (50% vs. 36%), more frequent use of sleep medication (4% vs. 7%), and more participants with a higher risk of having sleep apnea (17% vs. 24%) as compared to the medium sleep group. All other characteristics were similar between the groups.

### 3.2. Sleep Duration and Glycemic Traits

**Figure 1:**
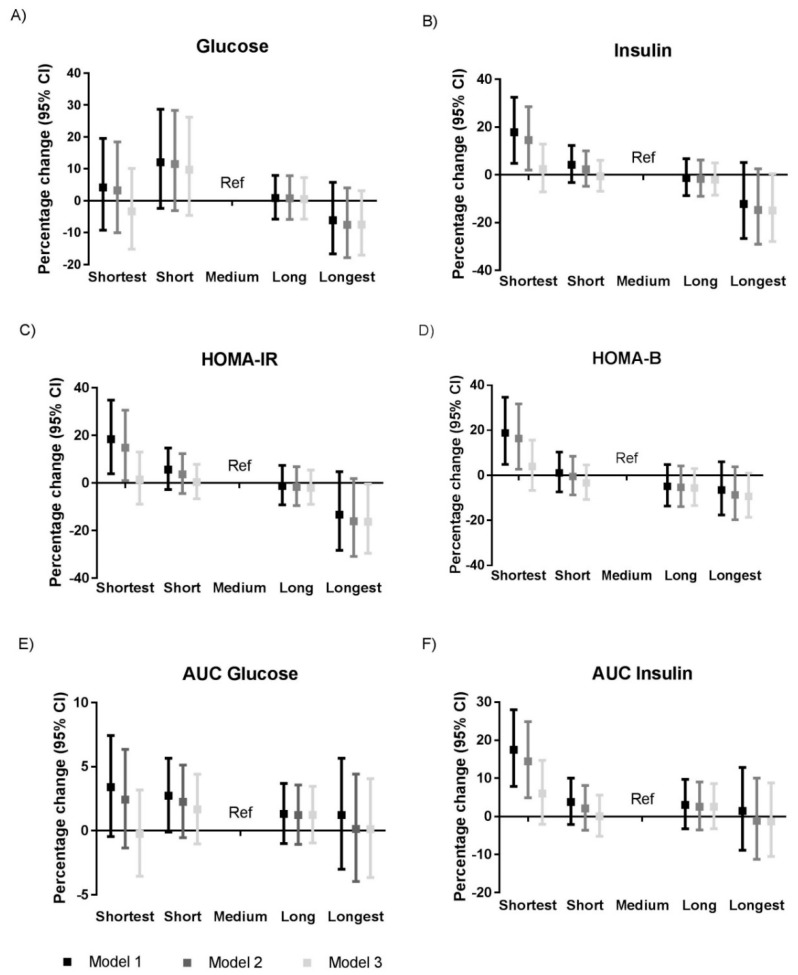
Associations between sleep duration and (**A**) fasting glucose, (**B**) fasting insulin, (**C**) HOMA-IR, (**D**) HOMA-β, (**E**) AUC of glucose, and (**F**) AUC of insulin. Results were based on analyses weighted towards the BMI distribution of the general Dutch population (*n* = 4672) and were derived from regression coefficients with 95% confidence intervals from linear regression analyses and expressed as percentage change in outcome measure, as compared to the medium sleep duration group as reference category. Model 1: Adjusted for age and sex; Model 2: Adjusted for age, sex, ethnicity, education level, smoking, alcohol intake, caloric intake, and physical activity; Model 3: Adjusted for Model 2 + risk of sleep apnea and BMI. Abbreviations: AUC, area under the curve; CI, confidence interval; HOMA-IR, homeostatic model of insulin resistance; HOMA-β, homeostatic model of β cell function; Ref, reference category. *n* = 4511 for the analyses with AUC glucose and *n* = 4586 for the analyses with AUC insulin.

In the analyses adjusted for age and sex (Model 1; explained variance up to 7.4), shortest sleep was associated with a 17.9% (95% CI: 4.8; 32.5%) higher fasting insulin, an 18.4% (95% CI: 3.9; 34.9%) higher HOMA-IR, an 18.8% (95% CI: 4.9; 34.7%) higher HOMA-β, and a 17.5% (95% CI: 7.9; 28.0%) higher AUC for insulin as compared to the medium sleep group (Figure 1 and Supplementary Table S1). When we adjusted for potential confounding factors (Model 2; explained variance up to 8.4%), shortest sleep duration was associated with a 14.5% (95% CI: 2.0; 28.6%) higher fasting insulin, a 14.9% (95% CI: 1.0; 30.7%) higher HOMA-IR, a 16.3% (95% CI: 2.7; 31.7%) higher HOMA-β, and a 14.5% (95% CI: 4.9; 24.9%) higher AUC of insulin. However, the associations between shortest sleep duration and HOMA-IR attenuated when analyses were adjusted for considered potential confounding factors (Model 2). When we additionally adjusted for BMI and risk of sleep apnea (Model 3; explained variance up to 29.5%) the other associations observed for shortest sleep duration disappeared. However, in Model 3, longest sleep was associated with a −16.3% (95% CI: −29.6; −0.4%) lower HOMA-IR as compared to the medium sleep duration group. The same attenuation of associations was observed when we adjusted separately for either BMI or the risk of sleep apnea (Supplementary Table S1: Models 3 and 4). 

### 3.3. Sleep Quality and Glycemic Traits

**Figure 2:**
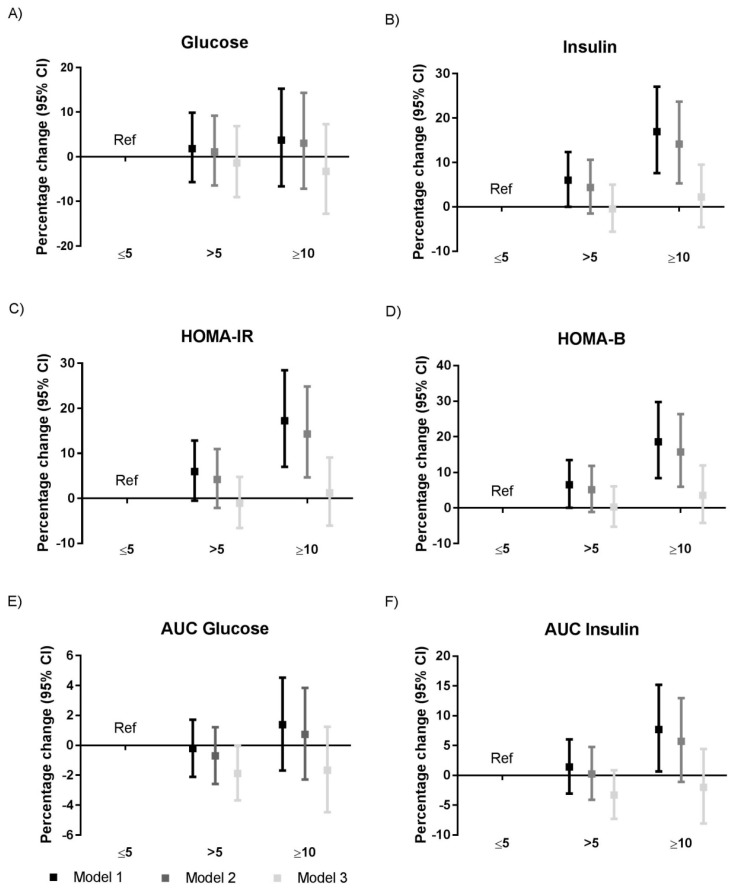
Associations between sleep quality and (**A**) fasting glucose, (**B**) fasting insulin, (**C**) HOMA-IR, (**D**) HOMA-β, (**E**) AUC of glucose, and (**F**) AUC of insulin. Results were based on analyses weighted towards the BMI distribution of the general Dutch population (*n* = 4672) and were derived from regression coefficients with 95% confidence intervals from linear regression analyses, using either a group with a PSQI total score of >5 or ≥10, and expressed as percentage change in outcome measure, as compared to the good sleep group as reference category. Model 1: Adjusted for age and sex; Model 2: Adjusted for age, sex, ethnicity, education level, smoking, alcohol intake, caloric intake, and physical activity; Model 3: Adjusted for Model 2 + risk of sleep apnea and BMI. Abbreviations: AUC, area under the curve; CI, confidence interval; HOMA-IR, homeostatic model of insulin resistance; HOMA-β, homeostatic model of β cell function; Ref, reference category. *n* = 4511 for the analyses with AUC glucose and *n* = 4586 for the analyses with AUC insulin.

The associations between sleep quality and glycemic traits are visualized in Figure 2 and Supplementary Table S2. We did find evidence for an association between poor sleep quality, as defined by a PSQI score of >5 and a 6.5% (95% CI: 0.0; 13.5%) higher HOMA- β in Model 1 (explained variance up to 7.1%) as compared to the good sleep quality group (PSQI score ≤5). However, when we adjusted for potential confounding factors in Model 2 (explained variance up to 8.0%), this association disappeared (percentage change 5.1% (95% CI: −1.1; –11.8%). In the age- and sex-adjusted analyses (Model 1), a PSQI score ≥10 was associated with a 16.9% (95% CI: 7.6; 27.1%) higher fasting insulin, a 17.3% (95% CI: 7.0; 28.5%) higher HOMA-IR, an 18.6% (95% CI: 8.4; 29.7%) higher HOMA-β, and a 7.7% (95% CI: 0.7; 15.2%) higher AUC of insulin as compared to the good sleep quality group. When we additionally adjusted for potential confounding factors in Model 2, we observed a 14.1% (95% CI: 5.3; 23.7%) higher fasting insulin, a 14.3% (95% CI: 4.7; 24.9%) higher HOMA-IR, and a 15.7% (95% CI: 6.0; 26.4%) higher HOMA-β in the group with a PSQI total score of ≥10 as compared to the good sleep quality group. However, all associations attenuated when we additionally adjusted for BMI and the risk of sleep apnea (Model 3; explained variance up to 29.2%). These associations attenuated in a similar manner when we adjusted the model for only BMI or the risk of sleep apnea (Supplementary Table S2: Models 3 and 4). 

### 3.4. Genetically-Determined Habitual Sleep Duration and Glycemic Traits

In order to assess whether the association observed between sleep duration and glycemic traits (Model 1 and 2) is causal, we performed an MR study. We found no evidence for an association between genetically determined total sleep duration, short sleep duration, and long sleep duration with glycemic traits using IVW-analyses (Table 2, Supplementary Table S3). The WME analyses were consistent with these results (Supplementary Table S3). However, we did find evidence for an association between total sleep duration and a higher HOMA-β (IVW-estimate: 0.08 (95% CI: 0.01; 0.014)). The estimate from WME analysis was consistent, and MR–Egger did not indicate the presence of directional pleiotropy in this analysis (Supplementary Table S3).

## 4. Discussion

In the present study, we performed a cross-sectional analysis in a middle-aged population of 4519 nondiabetic participants to determine the associations between habitual sleep duration and sleep quality with glycemic traits. When analyses were adjusted for age and sex, shortest sleep duration was associated with higher fasting insulin, higher HOMA-IR, higher HOMA-β, and higher AUC of insulin. Poorer sleep quality was associated with higher fasting insulin, higher HOMA-IR, higher HOMA-β, and higher AUC of insulin. However, all these associations disappeared after additional adjustment for BMI and the risk of sleep apnea. Furthermore, in the MR analyses, we, overall, did not observe associations between genetically-determined total, short and long sleep duration, and glycemic traits. Of interest, we found some evidence that longer genetically-determined sleep duration was associated with a higher HOMA-β.

Previous studies showed that both short and long sleep duration, or only short sleep duration were associated with a higher risk of obesity, body weight, insulin resistance, and diabetes mellitus [2,3,4,5,10,11]. We hypothesized that these differences in findings may be explained by confounding factors such as BMI and sleep apnea. In agreement, in the age- and sex-adjusted analyses, we observed associations between shorter sleep duration and higher insulin resistance, which disappeared when we additionally adjusted for BMI and the presence of sleep apnea. Our findings are in agreement with a study of Javaheri et al., who also observed an association between short sleep duration and insulin resistance, which disappeared after adjustment for obesity [18]. In line, in our MR study, genetically-determined sleep duration was, in general, not associated with glycemic traits, indicating that there is no direct effect of sleep on glycemic traits. However, it has been observed that there is a shared genetic component between sleep and anthropometrics [46]. Moreover, adiposity has been shown to be causally associated with an increased risk of diabetes and altered glycemic traits [16]. Therefore, we hypothesize that excess adiposity is a common cause for alterations in habitual sleep and insulin resistance, instead of being in the causal path of this association. 

An alternative explanation for these observations is that BMI/risk of sleep apnea is in the causal pathway between shorter sleep duration and increased insulin resistance. It was shown that sleep restriction reduces leptin levels and enhances ghrelin levels and thereby increases cravings for carbohydrate-rich foods, which may result in weight gain [47]. Another mechanism that links short sleep duration with insulin resistance might be via obstructive sleep apnea (OSA). It was demonstrated that patients with OSA showed increased insulin resistance compared to controls without OSA, independent of obesity [48]. These might indicate that, although obesity is known to effect insulin resistance [16], OSA may have an independent additional effect on insulin resistance, possibly via nocturnal hypoxemia [49]. A GWAS of Lane et al. on total sleep duration suggested that there is a shared genetic component between sleep duration and energy metabolism [46], which demonstrates that the genetic background of an individual may also interfere with altered sleep duration and energy metabolism. 

One of the strengths of this study is the use of residuals to determine sleep duration. As no clear cut-off for short and long sleep exists in literature, using residuals is a good way to harmonize grouping of sleep duration among studies. Moreover, the heterogeneity of future meta-analyses and systematic reviews might benefit from using this methodology. Another strength of this study is the extensive phenotyping of the NEO study. This enables us to correct for a broad range of possible confounding factors, such as ethnicity, physical activity, BMI, and risk of sleep apnea. In addition, we used the PSQI questionnaire, which is a widely used validated tool to assess sleep disturbances. A limitation of the present study is the cross-sectional design; therefore, we are not able to exclude reverse causation or residual confounding in this study. Another limitation of this study is the use of subjective sleep questionnaires to assess sleep duration, sleep quality, and the risk of sleep apnea. One of the drawbacks of self-reported data is the fact that these data are subject to recall bias. Moreover, there may be a measurement error resulting in nondifferential misclassification for the exposure. Therefore, the use of a sleep diary may improve reliability of the measures of habitual sleep. However, the PSQI questionnaire has been shown to be a reliable and validated tool to assess sleep dysfunction, and the Berlin questionnaire, used to assess the risk of sleep apnea, has been shown to be validated to detect mild and severe OSA in several populations [50,51]. The MR study has the advantage of being a highly efficient method that allows for usage of large sample sizes; however, although we used the largest GWAS to date, we cannot rule out the use of weak instruments. The 78 loci used in the current study only explained 0.69% of the variance in sleep duration. Moreover, the number of genetic instruments that were present in this current study, especially for long sleep duration, was minimal. Finally, the identification of the genetic variants as well as the MR study were both performed in cohorts of European ancestry, which hampers the generalizability to non-European populations. 

## 5. Conclusions

In summary, shorter sleep duration and poorer sleep quality were associated with higher insulin resistance, but these associations were dependent on BMI and the risk of sleep apnea. Both BMI and the risk of sleep apnea, as being potential confounding factors and/or factors in the causal pathway, thereby likely explain previous observed associations between adverse habitual sleep and an increased risk of insulin resistance, suggesting that these factors should therefore be considered in future studies. 

## Figures and Tables

**Table 1:** Characteristics of participants in the Netherlands Epidemiology of Obesity study, stratified by sleep duration (*n* = 4672).

Sleep Duration	Shortest	Short	Medium	Long	Longest
	0–5%	5–20%	20–80%	80–95%	95–100%
**Age (years)**	57 (5)	58 (5)	55 (6)	54 (6)	58 (6)
**Sex (% men)**	45	49	42	43	53
**BMI (kg/m^2^)**	27 (5)	26 (4)	26 (4)	26 (4)	26 (5)
**Ethnicity (% white)**	90	94	96	96	94
**Education (% high)**	39	41	50	48	36
**Smoking (%current)**	18	14	16	16	20
**Sleep medication (%)**	14	10	4	5	7
**Alcohol consumption (g/day)**	12 (3; 22)	11 (3; 22)	10 (3; 21)	9 (2; 21)	11 (1; 23)
**Physical activity (MET/h/week)**	25 (12; 44)	30 (16;50)	31 (17; 51)	32 (15; 52)	30 (16; 49)
**Sleep duration (h/day)**	5 (4; 5)	6 (6;6)	7 (7; 8)	8 (8; 8)	9 (9; 9)
**PSQI (total score)**	11 (9; 13)	7 (5; 9)	4 (3; 6)	3 (1; 4)	3 (1; 5)
**Sleep apnea (%)**	35	25	17	17	24
**Fasting glucose (mmol/L)**	6 (1)	6 (2)	6 (1)	6 (1)	6 (2)
**Fasting insulin (mmol/L)**	9 (6; 14)	8 (6; 12)	7 (5; 11)	7 (5; 11)	7 (4; 12)
**HOMA-IR**	2 (1; 4)	2 (1; 3)	2 (1; 3)	2 (1; 3)	2 (1; 3)
**HOMA-β**	28 (18; 42)	26 (18; 40)	24 (16; 37)	25 (15; 36)	25 (13; 37)
AUC Glucose *	6 (1)	6 (1)	6 (1)	6 (1)	6 (1)
**AUC Insulin ^#^**	47 (34; 62)	41 (30; 57)	38 (29; 53)	38 (30; 54)	41 (26; 61)

Abbreviations: AUC, area under the curve; BMI, body mass index; HOMA-β, homeostatic model of assessment of β-cell function; HOMA-IR, homeostatic model of assessment insulin resistance; kJ, kilojoule; MET, metabolic equivalents of task; NEO, Netherlands Epidemiology of Obesity; PSQI, Pittsburgh sleep questionnaire index. Results were based on analyses weighted towards the BMI distribution of the general Dutch population. Data presented as mean ± standard deviation (SD); proportion (%); median (25th–75th percentile). *, *n* = 4511; ^#^ *n* = 4586.

**Table 2:** Inverse-variance weighted estimates for sleep duration on glycemic traits.

		Total Sleep Duration		Short Sleep Duration		Long Sleep Duration
	SNPs	Estimate (95% CI)	SNPs	Estimate (95% CI)	SNPs	Estimate (95% CI)
**Fasting glucose**	54	−0.03 (−0.11; 0.06)	20	−0.09 (−0.19; 0.01)	5	−0.05 (−0.24; 0.13)
**Fasting insulin**	54	0.01 (−0.04; 0.07)	20	−0.01 (−0.11; 0.08)	5	−0.06 (−0.36; 0.23)
**HOMA-IR**	53	0.07 (−0.01; 0.15)	20	0.04 (−0.08; 0.15)	5	−0.01 (−0.47; 0.45)
**HOMA-β**	53	0.08 (0.01; 0.14)	20	0.09 (−0.01; 0.19)	5	0.09 (−0.26; 0.45)

Data presented as beta coefficients with 95% confidence interval per standard deviation increase in exposure.

## Supplementary Materials

The following are available online at https://www.mdpi.com/2077-0383/8/5/682/s1, Table S1: Associations between sleep duration and glycemic traits, Table S2: Association between sleep quality and glycemic traits, Table S3: Mendelian Randomization estimates for sleep duration on glycemic traits, Table S4: Genetic variants for sleep duration, Table S5: Genetic variants for short sleep duration, Table S6: Genetic variants for long sleep duration, Figure S1: Correlation plot of studied variables.
